# Factors influencing community engagement approaches used in *Aedes aegypti* management in Cairns, Australia

**DOI:** 10.1002/hpja.924

**Published:** 2024-09-25

**Authors:** Tammy Allen, Alan Crouch, Tanya L. Russell, Stephanie M. Topp

**Affiliations:** ^1^ College of Public Health, Medical and Veterinary Sciences James Cook University Cairns Queensland Australia; ^2^ Department of Rural Health University of Melbourne Melbourne Victoria Australia; ^3^ Australian Institute of Tropical Health and Medicine James Cook University Queensland Australia; ^4^ College of Public Health Medical and Veterinary Sciences, James Cook University Townsville Queensland Australia

**Keywords:** community engagement, dengue, high‐income country, mosquitoes

## Abstract

**Issue Addressed:**

An important part of preventing and managing *Aedes*‐borne mosquito disease outbreak risk is engaging the community. Research shows that high‐income countries tend to use top‐down measures for *Aedes* mosquito management, favouring educational approaches to engage the community over participatory approaches that actively involve and empower the community in addressing disease risk. Little is known about the reasons behind the use of these approaches and how they could be strengthened. This research explores the community engagement approaches used in *Aedes* mosquito management in Cairns, Queensland, Australia and the factors influencing the choice of these approaches.

**Methods:**

A case study design was used, drawing on two qualitative methods—key informant, semi‐structured interviews (*n* = 25), and a document review (*n* = 20). Thematic analysis was used to identify, analyse and attribute meaning from the data.

**Results:**

Various approaches were used to engage the community, including direct interaction through door‐to‐door inspections, broad outreach via mass media campaigns, and community participation in a novel mosquito replacement strategy. Factors influencing the choice of these approaches included government legislative responsibilities, research‐related ethical obligations, work norms within local government and public health units, the perceived importance of gaining community trust, constraints on workforce capacity, time and funding.

**Conclusions:**

There were multiple factors influencing the community engagement approaches used in this study. Resource constraints, institutional norms and prevailing attitudes and beliefs were identified as hindering the use of more empowering approaches to engaging the community. These barriers should be considered and addressed in the planning of *Aedes* mosquito management to better support community engagement in this setting.

**So What?:**

Community engagement is an important aspect of managing the *Aedes* mosquito disease threat. With the global increase in *Aedes* mosquito‐borne disease risk, these findings can help other at‐risk settings understand potential organisational impediments to engaging the community. This is particularly important when advocating for the inclusion of bottom‐up approaches in policy, and to ensure sufficient resources are allocated to strengthen community engagement in *Aedes* mosquito management.

## 1 INTRODUCTION

*Aedes* mosquito‐borne diseases, such as dengue fever, are an important global health challenge, with approximately 390 million dengue infections occurring worldwide each year.^1^ Although dengue fever disproportionately affects low‐ and lower‐ to middle‐income countries, sub‐tropical and tropical regions in high‐income countries such as the United States, Singapore and Australia experience *Aedes* mosquito‐borne disease risk. This risk is predicted to increase in high‐income countries due to factors such as global travel, climate change, and trade.^2^, ^3^

### 1.1 *Aedes*‐borne disease risk in Cairns, Australia

Australia is a high‐income country with a history of dengue fever outbreaks dating back to the late 1800s.^4^ Although the primary vector for transmitting dengue fever, *Aedes aegypti*, largely disappeared from Australia in the 1950s, this mosquito species remains in parts of Queensland, including the city of Cairns.^5^

**FIGURE 1:**
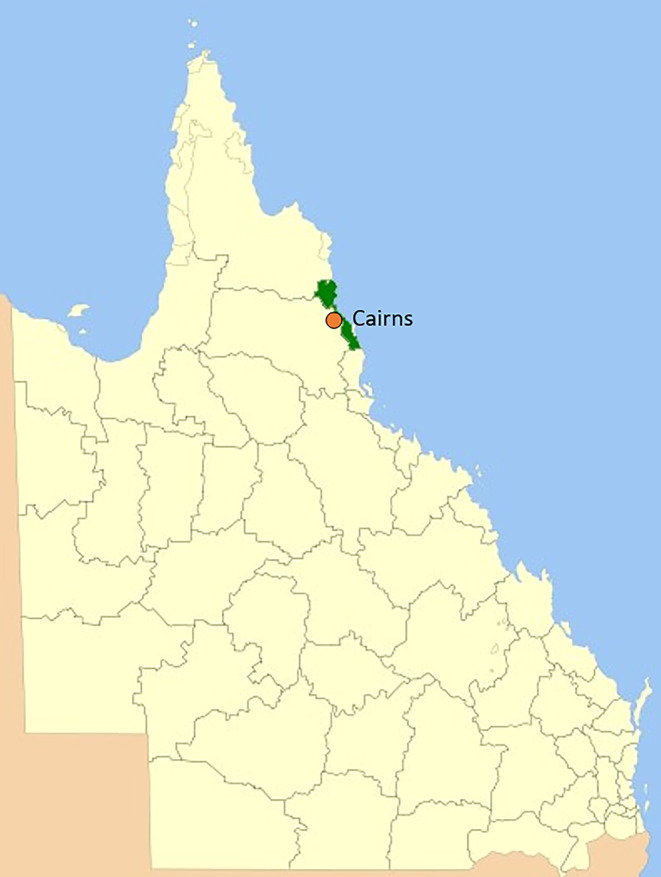
Map of Cairns Local Government Area. *Source*: Ref. https://commons.wikimedia.org/w/index.php?curid=3709702 (image adapted).

Cairns is a tropical, regional city located within the Cairns Local Government Area, in Far North Queensland, Australia^6^ (Figure 1). This region experiences a distinct wet season (November to March/April) and dry season (April to October).^7^ With an estimated population of 170 000, Cairns is a multi‐cultural community with 22% of residents born overseas and 10% of Aboriginal and Torres Strait Islander heritage.^8^ The region also boasts two world heritage‐listed sites—the Great Barrier Reef and Wet Tropics Rainforest, which attract millions of tourists each year.^9^

Over the last few decades, Cairns has experienced several large dengue outbreaks, including an outbreak in 1997–1999 (498 confirmed cases), in 2003/2004 (459 confirmed cases) and in 2008/2009 (938 confirmed cases)^10^, ^11^, ^12^ (Table 1).

**TABLE 1:** Dengue outbreak case numbers, Cairns (1990–2023).^10^, ^12^, ^13^, ^14^

Year	Outbreak case numbers
1990–1991	27^a^
1995	4
1996–1997	208^a^
1997–1998	12
1997–1999	498
2000	49
2002	2
2003	3
2003	5
2003–2004	459
2006	29
2008	1
2008	99^a^
2008–2009	900^a^
2012	7
2012–2013	146
2013	6
2013/2014	136
2014/2015	29
2015	2
2016–2023	No outbreaks with case numbers greater than >1

*Note*: Each line item represents a separate outbreak.

^a^ Multi‐city outbreak.

Multiple factors have contributed to *Aedes* mosquito‐borne disease risk in Cairns, including the presence of *Ae. aegypti*, climate influences, international travel and housing design (older houses lacking flyscreens).^15^, ^16^, ^17^ However, since 2016, there has been a notable decrease in dengue outbreaks in Cairns, largely attributable to the success of the Monash University, World Mosquito Program, which released *Wolbachia*‐infected *Ae. aegypti* in Cairns between 2011 and 2017. [Correction added on 29 October 2024, after first online publication: The starting year of World Mosquito Program in the preceding sentence has been corrected from 2011 to 2013.]. *Wolbachia* acts as a virus blocker in the mosquito to reduce *Aedes*‐borne disease transmission risk.^14^ Although Cairns is currently considered dengue‐free, vulnerabilities remain with the *Wolbachia* approach, including the potential loss of *Wolbachia* in the mosquito, evolutionary changes or resistance to virus strains, which could reduce the effectiveness of virus blocking.^18^ In addition, the ongoing threat of *Aedes albopictus* entering mainland Australia from the nearby Torres Strait region could result in this mosquito species establishing in both tropical and sub‐tropical regions, including Cairns.^19^

### 1.2 *Aedes* mosquito management

Managing *Aedes* mosquito‐borne disease risk in high‐income countries such as Australia, has traditionally involved vector control, mosquito surveillance, disease surveillance, and health promotion.^16^ In Queensland, *Aedes* mosquito management is a shared responsibility between the local government, who control nuisance and disease‐carrying mosquitoes, and the state government (Queensland Health), who prevent and respond to disease outbreaks, which includes *Ae. aegypti* control.^20^, ^21^ In Cairns, Queensland Health through the Tropical Public Health Services leads *Aedes* mosquito management.^22^

### 1.3 Community engagement and *Aedes* mosquito management

Engaging the community is an integral part of *Aedes* mosquito management, particularly given the *Ae. aegypti* lives and breeds in and around people's homes.^23^ Community engagement can be defined as the *‘process of working collaboratively with and through groups of people affiliated by geographic proximity, special interest, or similar situations to address issues affecting the wellbeing of those people*.’^24^ Approaches to engaging the community can vary depending on the purpose of engagement, from informing, to involving, collaborating, and empowering the community.^25^ The World Health Organization recommends that authorities collaborate with key stakeholders and the local community, utilising local knowledge, skills and resources, to plan and implement vector control strategies that encourage and support community ownership.^23^ A recent review of community engagement approaches used in *Aedes* mosquito management, specifically in high‐income countries, found that authorities tended to use government‐led or ‘top‐down’ approaches to vector control, engaging the community primarily through education to promote behaviour change.^26^ Although ‘top‐down’ approaches to *Aedes* mosquito management can be effective, particularly during outbreak response, they can also be resource‐intensive, difficult to sustain, and can lead to community apathy.^27^ The incorporation of participatory or ‘bottom‐up’ engagement approaches can enhance community ownership and responsibility for vector control. However, these types of engagement approaches are known to take time to establish and sustain.^23^, ^27^

To better understand the challenges and opportunities for engaging the community in *Aedes* mosquito management, it is important to understand the factors that have influenced the choice of community engagement approaches.

This study explores the community engagement approaches used in *Aedes* mosquito management and the factors influencing the choice of these approaches. We use *Cairns*, *Queensland*, *Australia*, as a case study for this exploration.

This qualitative case study had two objectives:

1. To explore the community engagement approaches that have historically been used in *Aedes* mosquito management in Cairns, Australia.
2. To understand the factors that have influenced the choice of these approaches.

## 2 METHODS

### 2.1 Study design

This study uses a descriptive, case study design drawing on prominent, contemporary case study theorists including Simons,^28^ Merriam^29^ and Yin.^30^ Simons defines case study design as an ‘*in depth exploration of the complexity and uniqueness of a particular project, policy or program in a real life context*.’^28^ Case study design is particularly useful for understanding a system from a participant's perspective.^30^ This research design enables in‐depth exploration of the community engagement approaches used in *Aedes* mosquito management and what influenced the use of these approaches.

The case unit for this research was defined by *Aedes* mosquito management ‘system’ and the community engagement approaches used within this system. The case boundary was defined by the geographic limits of the Cairns Local Government Area^31^ (Figure 1). Case boundaries were extended to accommodate variations in the source of funding and management of the *Aedes* mosquito management programs (e.g., World Mosquito Program) that went beyond the geographical boundary.

### 2.2 Data collection and participants

Two qualitative data collection methods were used for this research—semi‐structured, key informant interviews and an in‐depth document analysis. Data collection occurred between 2019 and 2023.

Semi‐structured, key informant interviews enabled exploration of participants' experiences and perspectives of engaging the community in *Aedes* mosquito management. Purposive sampling drawing on investigator knowledge and publicly available information was used to select suitable participants for this study. This was supplemented by snowball sampling to extend and deepen understanding through referrals to previously unidentified, but knowledgeable individuals.^32^ Inclusion criteria included current or previous (1990–present) involvement in *Aedes* mosquito management in Cairns. For this study, *Aedes* mosquito management is defined as the ‘surveillance, prevention and control of *Aedes* mosquitoes (specifically *Ae. aegypti*) and mosquito‐borne disease threats resulting from these mosquitoes in Cairns.’^21^

At the time of data collection, there were three key organisations involved in *Aedes* mosquito management in Cairns:

1. State Government, Queensland Health, Tropical Public Health Services.
2. Local Government, Cairns Regional Council, Vector Control Unit.
3. Monash University, World Mosquito Program.

We contacted each organisation and extended an invitation to those who fit the inclusion criteria, to participate in an interview. In total, 25 people were interviewed for this study (Table 2).

**TABLE 2:** Organisation and number of interviewees.

Organisation	Number of interviewees
Tropical Public Health Services	10
Cairns Regional Council	1
Monash University, World Mosquito Program	14
Total interviewed	25

In addition, 20 documents (published and unpublished) were reviewed for information on *Aedes* mosquito management approaches (objective one) and any institutional factors related to the community engagement approaches used (objective two). This data collection method also aided in tracing key decisions leading up to pivotal changes in community engagement approaches and helped in triangulating the data collected through the semi‐structured interviews. The process used to source the documents involved targeted searches of relevant authority's websites and relevant documentation requested from key informants during the interview process (Supporting Information S1).

### 2.3 Data analysis

Thematic analysis was used to identify, analyse and attribute meaning from the data collected. Each interview was transcribed verbatim and imported into NVivo12+. A combination of inductive and deductive coding was used to analyse the interview data and documents. This ensured analysis remained linked to the data as well as responding to the study objectives. Deductive analysis drew on the IAP2 Public Participation Spectrum©^25^ and Community Empowerment Domains^33^ to help identify the different types of community engagement approaches used (objective one). Scott's Institutional Analysis Theory, which examines elements within an institution that influence the behaviour of individuals and organisations,^34^ informed our organisation of the factors influencing the selection of these approaches (objective two). These factors were then grouped into four domains: *Regulatory* (laws and regulations), *Normative* (the institutional norms that shape community engagement approaches), *Social‐cognitive* (attitudes and beliefs towards engaging the community) and *Resources* (the resourcing factors that influence community engagement).

### 2.4 Ethics statement

This study was approved by the Townsville Hospital and Health Service Human Research Ethics Committee, Australia (HREC/2019/QTHS/53053). Interviews and subsequent analysis followed the relevant guidelines and regulations as stipulated in the ethics approval. Informed consent was obtained from all participants. Participation in the study was voluntary and confidential. Standards for Reporting Qualitative Research were used as a guide for reporting on this research.^35^

## 3 RESULTS

The findings are organised into two sections. First, we describe the community engagement approaches used by the three agencies involved in *Aedes* mosquito management in Cairns (objective one). Second, we examine the key factors influencing the choice of community engagement approaches used (objective two), described under the four domains of regulatory, normative, cognitive, and resource factors. Quotes are attributed to individuals from either—Cairns Regional Council (CRC), Tropical Public Health Services (TPHS), or the World Mosquito Program (WMP), and a sequential ID.

### 3.1 Community engagement in *Aedes* mosquito management in Cairns

#### 3.1.1 Tropical Public Health Services (formally Tropical Public Health Unit/Network)

Community engagement has been a core part of *Aedes* mosquito management implemented by the Tropical Public Health Services (TPHS) notably since the establishment of the public health unit in Cairns, in 1992,^36^ and the subsequent development of the first Queensland *Dengue Fever Management Plan* in 1994.^22^ The first Dengue Fever Management Plan provided guidance on vector control, vector surveillance, disease surveillance, and health promotion, with a focus on raising awareness of what the community can do to reduce mosquito larval habitats.^22^ During the 1990s, community engagement approaches to support *Ae. aegypti* control were operationalised primarily through three departments at TPHS—public affairs (typically 1× person), health promotion (typically 1× person), and a small vector control/environmental health team. These departments worked together to develop and implement a range of broad community education approaches, including a ‘*Stop Dengue Now Aye*’ television and radio campaign; printed materials (factsheets and pamphlets) with the key messages ‘*Tip them out, Store them dry, Throw them out*’; and a ‘*Flozzie the Mossie*’ school‐based education program delivered to select Cairns' primary schools, in conjunction with the Department of Education and the Cairns Rotary Club.^37^

A pivotal change to community engagement occurred during a large dengue outbreak in 1997 (498 confirmed cases).^10^ Despite concerted efforts to educate the community through the approaches outlined above, TPHS staff found extensive larval habitats in and around people's homes.^22^ During this outbreak, a decision was made to establish a dedicated, state government‐funded and managed, Dengue Action Response Team (DART) housed at TPHS. The DART initially consisted of three specialist vector control officers focused specifically on dengue outbreak control.^22^ The DART was primarily a top‐down vector control model, engaging residents via door‐to‐door inspections to seek permission to conduct vector control in and around their properties. During this same outbreak, the community was engaged through mass media communications. For example, TPHS public affairs staff developed a new public relations campaign focused on elevating risk perception, with a ‘dengue watch level’ communicated to at‐risk suburbs and a ‘dengue warning level’ for suburbs with confirmed cases. These messages were communicated through local newspaper, television, and radio advertisements. Print materials (pamphlets, bin stickers, fact sheets and fridge magnets.) were also disseminated to the general population via the post and the DART, and to high‐risk settings (e.g., garden centres, tyre yards, backpacker hostels and construction sites). Regular media briefings and press conferences were also used to communicate key messages throughout the outbreak.^38^, ^39^

The next decade (1997–2007) saw the continuation of existing and new community engagement approaches. The DART, now a permanent fixture at the TPHS, continued to engage residents through door‐to‐door inspections leading up to the wet season (December–March) and during outbreak response. A large multi‐city dengue outbreak in North Queensland in 2003/4 (Cairns—459 confirmed cases)^11^ saw the TPHS DART introduce a new top‐down, ‘lure and kill’ vector control strategy, which entailed placing traps on resident's properties.^11^ This outbreak also led to a ‘Dengue Blitz’ media campaign—a joint community service initiative between TPHS public affairs staff and the local media outlet (Cairns Post) promoting the key messages of getting rid of larval habitats and using insect repellent.^40^ Shortly after this outbreak (2006), a new ‘*Flozzie the Mossie*’ community‐wide prevention campaign was developed using mass media (television and radio) and print materials (posters, pamphlets and stickers) targeting residents and high‐risk settings with the key message ‘*if you stop the mozzie breeding, you can stop the disease*.’

Community engagement approaches were enhanced in 2008, in response to a large dengue outbreak in Cairns and surrounding areas (2008–2009; 938 confirmed cases).^12^ This explosive outbreak led to a swift decision by the Department of Health, Brisbane, to develop a new outbreak awareness campaign, ‘*Defend Against Dengue*.’ ‘*Defend Against Dengue*’ aimed to promote protective behaviours, such as seeing a doctor if symptomatic, wearing insect repellent, and getting rid of larval habitats. The DART continued to use top‐down approaches such as treating larval habitats with the insect growth regulator methoprene and labour‐intensive indoor residual spraying, requiring resident's approval.^12^ They also distributed free rubbish tip passes and collaborated with the local state emergency services volunteer group to disseminate surface spray and information to residents, and used a trailer to eliminate large, unwanted items from high‐risk properties. The communicable disease control staff alerted doctors to dengue symptoms and promoted protective behaviours among confirmed cases to prevent the spread of the disease.

After the 2008/2009 dengue outbreak, the Defend Against Dengue campaign remained operational under the central administration of Queensland Health.^41^ A TPHS health promotion professional focused on addressing key barriers to the community taking preventative action. For example, they supported a community‐led project trialling the placing of bins (skips) across a suburb, to encourage local residents to dispose of large unwanted items such as containers, tyres and white goods. The local school was encouraged to participate, and volunteers were available to assist those unable to clean up their own backyards.

In 2012, community engagement approaches experienced a significant setback due to the cessation of the dedicated TPHS health promotion position and the accompanying local project funding for dengue prevention. This change followed a state government decision to downsize Queensland Health's non‐clinical workforce.^42^ From this point, the DART continued to engage the community through routine door‐to‐door inspections, ad hoc school education sessions and community events. By 2019, some of these engagement approaches ceased, due to the success of the WMP. Notably, one of the DART members detailed how, although the DART continued to respond to imported dengue cases (conducting interior spray in a case house), residents were no longer routinely engaged through door‐to‐door inspections, and therefore this work was no longer a priority for TPHS.

#### 3.1.2 Local Government, Cairns Regional Council

The Cairns Regional Council (CRC), vector control team played a role in engaging the community in *Aedes* mosquito management in Cairns. Despite having legislative responsibilities for *Aedes* mosquito control, the establishment of the DART at TPHS in 1998 resulted in the CRC supporting rather than co‐leading *Aedes* mosquito management in Cairns.^20^, ^21^, ^22^ The council engaged the Cairns community through routine and outbreak door‐to‐door inspections, following the lead of the DART at TPHS. Given the CRC's legislative powers to fine properties owners, the vector control team were specifically tasked to focus on high‐risk properties and workplaces (e.g., garden centres, tyre yards, backpacker hostels and construction sites) to inform property owners/managers of source reduction measures and of their legal responsibility to prevent mosquito breeding.

This engagement continued until a pivotal change occurred in 2019 with the success of the World Mosquito Program (as previously described). In the interviews, a CRC vector control team member described how they were now doing fewer door‐to‐door inspections, focusing their efforts on addressing nuisance or pest mosquitoes, not related to the *Aedes* mosquito. They described continuing to promote dengue information on CRC's website, and conducting education, by request, to school children in Cairns.

‘We do a little bit of education. Over the years, we've done school programs and things like that where we offer information sessions … showing them how we do light traps …, and some basic mosquito identification stuff’ (CRC1).

#### 3.1.3 World Mosquito Program

Community engagement was integral to the Monash University, World Mosquito Program (previously Eliminate Dengue), a new *Aedes* mosquito management program introduced to Cairns in 2008. Funded by the Gates Foundation and the Queensland Government, the World Mosquito Program's primary objective was to pilot a world‐first biological technique inoculating *Ae. aegypti* with *Wolbachia*, to reduce the mosquito's capacity to transmit diseases, such as, dengue to humans, and hence releasing these mosquitoes into the wild mosquito population.^14^

Two geographically distinct communities—Gordonvale and Yorkeys Knob were identified as the first sites in Cairns for the *Wolbachia*‐infected mosquitoes to be released in 2011. Prior to this, the World Mosquito Program spent 2 years (2008–2010) extensively engaging the community to understand dengue knowledge and attitudes, and perceptions and concerns related to the release of *Wolbachia*‐infected mosquitoes into the community.^43^ Engagement approaches included surveys, interviews, focus groups, community meetings, community events and having a local community reference group. The information garnered during this pre‐release phase, helped inform mosquito release approaches, including the decision to seek written consent from each household for the release of *Wolbachia* mosquitoes in the pilot suburbs. Community engagement continued throughout the *Wolbachia* mosquito pilot release phase (2011–2012). Approaches included temperature testing with surveys, newsletter updates, media updates and reference group meetings.

‘We did what was referred to as temperature testing with surveys, that we would randomly knock on doors and ask people if they're aware of, had they been experience more mozzies, did they know about the project? How did they feel? Did they want any more information?’ (WMP3).

Buoyed by the successful release of *Wolbachia* mosquitoes in Gordonvale and Yorkeys Knob, the World Mosquito Program expanded to include more small‐scale releases in various suburbs of Cairns between 2011 and 2014. Community engagement approaches were similar to the pilot phase, including gaining permission from residents, community meetings, letterbox drops containing project updates, media engagements and paid advertising to keep residents informed.^14^, ^44^ In addition, a city‐wide reference group was established comprising representatives from tourism, government (local government, health and education), and local community leaders to support the releases.

From 2015, until the program's conclusion in 2017, the World Mosquito Program in Cairns adopted a Public Acceptance Model which supported the scaling‐up of community engagement for larger mosquito releases across the rest of the city, including a shift away from attaining individual consent. Engagement approaches included:

1. Awareness‐raising through media, community events and information kiosks;
2. Involving primary school children in mosquito releases through the *Wolbachia* Warrior Program;
3. Promoting a system for participants to contact the program with concerns or to opt in or out of participating in the program
4. Utilising a community reference group to review community engagement approaches.^14^

### 3.2 Factors influencing the choice of community engagement approaches

#### 3.2.1 Regulatory factors

Regulatory factors, including the legislative responsibility of residents, and the powers that authorities work under, influenced the community engagement approaches used by the TPHS DART and the CRC vector control team in Cairns.

##### Government responsibility

Under the Queensland Public Health Regulation (2018) ‘*A relevant person for a place must ensure water or another liquid that has accumulated at the place is not a breeding ground for mosquitoes*.’^20^ Consulting with residents and business owners about their responsibility under this regulation was a key part of engaging residents during property inspections. In particular, the CRC vector control/environmental health staff were delegated the authority to ensure compliance with this legislation, which shaped who (primarily high‐risk settings) and how (through inspections) they engaged with the community. For example, property owners can be issued with a public health order by either authority (CRC or TPHS) to clean up their property, with the CRC having the power to impose a fine, if necessary.^20^

‘If we get to the point where we serve a public health order, we will follow it through until there's no mosquito breeding on the property’ (CRC1).

Although residents are responsible for controlling mosquito breeding on their properties, the Queensland Public Health Act (2005) stipulates that vector control activities can be supplemented by Queensland Health (TPHS) and/or local government (CRC) if there is a risk of, or actual, disease outbreak. These government‐led activities are run under an Authorised Prevention and Control Program, which enables authorities to conduct vector control on behalf of residents. It requires brief engagement with a resident if they are at home to seek permission to inspect and treat private properties (surrounding the house) and/or communication with a resident if they are found to be breeding mosquitoes.^45^ In the interviews, one of the DART members described how this legislation enhanced efficiency, particularly during outbreak response, when time and resources were stretched. They interpreted these regulatory provisions as grounds for minimising their engagement with residents, if required, to ensure they could complete the property inspection.

‘It [legislation] gave you access, it had nothing to do with convincing people, they didn't really have a say’ (TPHS5).

#### 3.2.2 Normative factors

There were several institutional norms driving community engagement approaches, including authorities' views on who should be leading vector control, and perspectives on why the community should be engaged.

##### Authority‐led vector control

In addition to the regulatory framework (as previously described), the firmly held view that government‐led (top‐down) intervention was necessary, particularly during outbreak response, influenced the DART's approach to community engagement. Some DART members highlighted the importance of expert‐led outbreak response to efficiently identify, access, and chemically treat real and potential larval habitats. These norms led to less time spent engaging residents during outbreak response, with the focus of engagement on seeking permission from residents to carry out this work. Conversely, outside of outbreaks, although residents were encouraged to get rid of larval habitats themselves, some of the DART members highlighted the importance of having government guidance (for the reasons stated during outbreak response), rather than the community leading or facilitating vector control strategies.

‘I don't think it's a very good idea to let everyone go, without someone monitoring. Everyone can do the basics, but I don't think it's a good idea getting everyone running around doing their own thing’ (TPHS1).

Furthermore, the belief held by state government (TPHS) that they should be leading mosquito management efforts in Cairns, shaped local government (CRC) vector control's role in engaging the community. Despite CRC having legislative responsibility for managing disease‐carrying mosquitoes, the establishment of the DART in 1998 led to a shift in CRC's vector control operational responsibilities, focusing their engagement efforts primarily on high‐risk properties to ensure legislative compliance.

‘Mosquito control is a local government public health risk, so the unintended consequence of our DART was that Cairns City Council [CRC] said, oh, OK, if you're gonna have a DART, then we'll disengage in the mosquito because it's clearly a state government thing’ (TPHS4).

##### The ethical obligation to engage community

Conversely, different normative factors drove some of the decisions related to the community engagement approaches used in the World Mosquito Program, particularly during the pre‐release phase. Although government regulatory approvals did not specifically stipulate a requirement to engage the community, several of the World Mosquito Program staff interviewed, along with previous research conducted in this area, highlighted the ethical obligation of seeking and sustaining community support for the World Mosquito Program to be successful.

‘The permit from the APVMA [Australian Pesticides and Veterinary Medicines Authority] was enough to release it. It didn't need community support, as in, it didn't require a regulation and a tick box. It was an extra requirement of the research project was we needed the community … to make the project work; we needed the support of the community’ (WMP3).

#### 3.2.3 Social‐cognitive factors

Social‐cognitive factors also influenced the choice of community engagement approaches used across the three organisations, including perceptions of community apathy, the need to gain trust and credibility, and leadership beliefs and attitudes.

##### Perceived community apathy

In the interviews, staff across the three organisations described perceived misconceptions by the community of where the *Aedes* mosquito can breed and, for some parts of the community, a lack of concern for mosquito breeding as a potential health issue. This perception led to authorities focusing their engagement efforts on awareness‐raising and community education. Perceived apathy by the community also led to frustration when interacting with the community, which at times, influenced the priority that the DART and the CRC dedicated to engaging the community, particularly during the dengue outbreak response.

‘there's that percentage of people that don't tend to have much of a care factor … They don't see it as a significant thing, even though we try to explain’ (CRC1).

‘I was telling people, “Watch out for your palm fronds, or anything that can hold water etc.” And I had people saying, “Oh yeah, well look, there's a big swamp down the back there. Why don't the council come out and fill that?” Well, it's just ignorance’ (WMP7).

‘I find the adults have almost no idea except they might say potplant bases, bird baths—or they say “I cleaned that yesterday or the day before” … more worried about being caught or in trouble than solving the problem. More defensive. A lot of people saying you won't find anything in our yard?’ (TPHS1).

##### Gaining trust and credibility

The relative need to gain trust and credibility with the community also influenced investment in community engagement, particularly for the World Mosquito Program. As this was a new program introducing a biological control measure in and around people's homes, community engagement was important as an operational imperative to avoid challenges to its ground‐breaking work.

‘Cairns was a bit like a bank in the sense that you know how a bank has to engender trust. Cairns was very methodical in how it did its research and its approach because it needed to establish credibility’ (WMP5).

As part of this, the World Mosquito Program staff were conscious of addressing the community's expressed uncertainties and safety fears.

‘Because it was predominantly about safety. You know, “I'll support you if you can convince me or assure me that it's safe for myself, my family, my pets, and the environment”’ (WMP3).

Gaining trust was also important for the TPHS, particularly during outbreak response when the DART was needed to enter people's private properties and conduct vector control. Interviews pointed to the fact that residents who had multiple larval habitats on their properties sometimes lacked trust in the government, which posed a challenge when assisting residents to comply with the regulation for prohibiting mosquito breeding^20^ and in some (rare) cases led to the resident being fined.

‘They just locked their doors and then you can't get in … you will always have that, anti‐government “what will they know about that, they know more than we know?”’ (TPHS3).

##### Leadership beliefs and attitude

Leaders' attitude towards engaging the community was a key influential factor in the priority placed on this work as part of *Aedes* mosquito management in Cairns. Some leaders were found to have a positive influence on advocating for the importance of community engagement. For example, the inclusion of health promotion as a core component of the first Dengue Fever Management Plan in 1994 was attributed to the support and influence of the TPHS Director at the time. The Dengue Fever Management Plan described the importance of community education to raise awareness of dengue fever prevention and protective behaviours.^22^ Recognition of health promotion in strategic guidance supported the instigation of awareness‐raising campaigns, which were developed by TPHS during the 1990s.

Several staff members from the World Mosquito Program reported that leadership were supportive of and committed to prioritising community engagement, especially during the pre‐release phase. One staff member noted that a World Mosquito Program scientific leader actively engaged with the community, which significantly influenced the emphasis placed on community engagement throughout this program.

‘*WMP leader* was always community first with him. He understood the value of engaging the community. And if you don't have that at the top, you are stuffed’ (WMP4).

‘They're *WMP leadership* truly involved. I think it all comes down to good leadership and good management, in dealing with the community’ (WMP3).

However, some in leadership roles were more cautious of the benefits of community engagement and hence the priority that should be placed on this work. For example, one DART member in a leadership role referenced the case of Singapore, where despite an intensive focus on community education and social mobilisation approaches, large dengue outbreaks have still occurred. This attitude underpinned some of the norms (as previously described) related to the DART conducting vector control on behalf of residents and at times minimally engaging with residents.

‘I'm almost like those climate change skeptics, I'm very skeptical of social mobilization and citizen science, it's all the buzz words’ (TPHS5).

#### 3.2.4 Resourcing

Several resourcing factors, including workforce capacity and funding, influenced the extent to which the community was engaged across the three organisations.

##### Workforce capacity, time and funding

Limited funding, time and capacity influenced the type of approaches used to engage the community. For example, despite recognition of the importance of health promotion in the Queensland Dengue Fever Management Plan (1994), there was still limited funding available from either authority (TPHS or CRC) to implement community awareness campaigns in the 1990s. This meant a reliance on free media advertising via community service announcements and external sponsorship, for example, Aeroguard (a mosquito repellent company) to engage the broader community in promoting preventative behaviours.^40^

As outbreaks increased, reactive funding facilitated the development and implementation of mass media campaigns during periods of heightened dengue risk. For example, the ‘*Screen it up, slap it on, tip em*’ *out’* education campaign in the 1990s was developed using funding available to respond to an outbreak in the nearby city of Townsville.^36^ By the end of the 1990s, recurrent, annual funding was secured to screen mass media campaigns throughout north Queensland. Outbreak funding continued to be used to update or develop new campaigns. For example, a multi‐city outbreak in 2003 was a catalyst for the development of the ‘Flozzie the Mossie’ television and radio campaign, and a large dengue outbreak in 2009 triggered the development of a ‘*Defend Against Dengue*’ social marketing campaign.

In addition, lack of funding as well as limited capacity and competing priorities were described by TPHS vector control staff as influencing the extent to which the community was engaged beyond door‐to‐door inspections. In the interviews, several DART members described having other responsibilities such as *Ae. aegypti* surveillance, research, training, supporting CRC vector control staff, and implementing the *Ae. albopictus* Elimination Program in the Torres Strait. This hindered the priority given to engaging the community. In addition, TPHS described a lack of funding and time as key barriers to involving the community in broader vector control practices.^16^

‘Every strategy is going to have a cost to it. At the moment, you don't give sprays and traps *to the community*. In order to give people tools, you need funding. In order to get funding, what do you think needs to happen? In order for more bottom‐up to happen, we need money’ (TPHS5).

‘We get all the time: “Will you guys let us know what is in that trap?” The bottom line is: “probably not.” We don't have someone sitting there who could do that. If you had someone sitting there who was community engagement, otherwise what can do?’ (TPHS1).

The CRC's capacity to engage the community in *Aedes* mosquito management was also limited by a lack of a dedicated workforce. At the time of the interviews (2019), a CRC team member described that recent funding cuts had led to a downsizing of their vector control team from 6x vector control staff and an environmental health officer, to 1× environmental health officer, and 1× vector control officer. This small team had to prioritise their vector work, which was now focused on addressing nuisance mosquito complaints and fogging to reduce Ross River Virus risk.

Conversely, the World Mosquito Program had a dedicated workforce and funding to engage the community throughout the program (2008–2017), which influenced the extent to which the community was engaged, particularly prior to the release of the *Wolbachia* mosquitoes. This program was funded by the Bill & Melinda Gates Foundation (through the Grand Challenges in Global Health Program).^46^ For the first 2 years (pre‐release, 2008–2010), the World Mosquito Program employed a medical anthropologist to engage the community and a communications professional to develop communication materials to support community engagement. In the interviews, one of the World Mosquito Program members described how the medical anthropologist was instrumental in garnering community support and influencing the engagement approaches developed during this time. Once the mosquitoes were released, the World Mosquito Program continued to employ community engagement/communication specialists until the program finished in 2017. Although there was a community engagement workforce, given the mosquitoes were being released on a larger scale than the pilot communities, the community engagement approaches changed to reflect the practicalities of the workforce engaging the community on a larger scale, hence the adoption of the Public Acceptance Model community engagement framework (as previously described).^14^

## 4 DISCUSSION

This case study draws on historical document analysis and key informant interviews across three organisations to offer valuable insights into the community engagement approaches implemented in Cairns, a city with a history of dengue outbreaks. Additionally, this study sheds light on the key factors influencing the choice of these approaches. We reflect on these two objectives in the following discussion.

In the first instance, our research revealed various approaches to engaging the community in *Aedes* mosquito management, over the last 30 years. There was a notable focus on using mass media campaigns and door‐to‐door inspections as key approaches to inform residents of dengue fever prevention and protective measures, and to encourage community mobilisation, particularly during outbreak events. These approaches typically align with the informing and consulting levels of engagement, as described by well‐known community participation frameworks such as the IAP2 Public Participation Spectrum©.^25^ The mass media campaigns described in this study were important to communicate risk, raise awareness and promote responsibility to a wide audience, particularly during outbreak response.^22^ However, relying on this approach to achieve sustainable behaviour change requires caution.^47^ For example, an evaluation of the Queensland Health ‘Flozzie the Mossie’ Campaign (2007) screened in Cairns, showed a high recall of campaign messages and knowledge of dengue prevention behaviours, yet nine out of 10 of those surveyed admitted to still having one or more items of potential breeding sites in and around their homes.^48^ The emphasis on mass media campaigns was congruent with approaches commonly used in *Aedes* mosquito management in other high‐risk settings.^26^ Mass media campaigns should be complemented by approaches that address barriers to taking action, and actively involve the community in the process of identifying and getting rid of potential larval habitats.^23^, ^49^ Indeed, the study described examples of authorities working with community volunteers to disseminate mosquito repellent (2008 outbreak) and supporting a community‐led ‘skip‐bin’ event (2010), demonstrating elements of actively involving the community in *Aedes* mosquito management©.^25^ However, these approaches were found to be ‘one‐off’ events not sustained over the period studied.

In the second instance, this case study explored why these community engagement approaches were used. Firstly, it is pertinent to note that there were fundamentally different purposes for engaging the community across the three organisations, shaping the type and extent of community participation approaches used. For example, the primary purpose for the WMP engaging the community was to gain and sustain the community's trust and support for the *Wolbachia* mosquito releases. Supportive leadership, normative values (ethics) and workforce capacity underpinned the use of these approaches. Similar findings were described by Kolopack et al., whose research also found internal leadership, resourcing (time, expertise and funds) and commitment to meaningful engagement (ethics) shaped the success of community engagement in the WMP.^50^ Conversely, the emphasis of local and state government authorities on more top‐down approaches was driven by legislative requirements, organisational norms, and the perception that community members did not prioritise mosquito control, assuming it to be a government responsibility. These perceptions were congruent with a Queensland Health campaign evaluation survey (as previously described) of Cairns residents (2004 and 2007), which found prevailing assumptions that *Ae. aegypti* also breeds in swamps and rivers, hence being the responsibility of government to control, and an assumption that government, primarily local government, were responsible for controlling mosquitoes during an outbreak.^47^ This assumption may have stemmed from residents observing both TPHS (state government) and CRC (local government) conducting vector control on their behalf (under the Authorized Prevention and Control Program) during the numerous dengue outbreaks in the 1990s and early 2000s. In addition, as dengue was not endemic in Cairns and as dengue‐related deaths rare, this may have also contributed to the community's perceived low level of threat towards dengue and likelihood of taking preventative action.

While good practice guidelines for *Aedes* mosquito management recommend actively involving the community in planning and implementing locally accepted strategies,^23^, ^49^ this study identified multiple constraints to doing this, including lack of time, funding, and capacity. Recognising and prioritising ways to address these barriers is important when planning *Aedes* mosquito management to optimise the likelihood of community participation in reducing disease risk. With the global increase in *Aedes* mosquito management risk, these findings can help other at‐risk settings understand potential impediments to community engagement. This is particularly important when advocating for the inclusion of bottom‐up approaches in planning and policy, and for ensuring sufficient resources are allocated to strengthening community engagement in *Aedes* mosquito management.

### 4.1 Study limitations and future research priorities

This research provides unique insights into *Aedes* mosquito management engagement approaches used by authorities in Cairns, since the 1990s. Our research primarily focused on understanding the perspectives of authorities regarding community engagement. Therefore, we did not capture the community's views on their involvement in *Aedes* mosquito management. This is a notable limitation and warrants investigation as a future research priority. We also draw attention to the timeframe covered by this research (30 years), which presented challenges in finding informants, particularly from local government, with knowledge from the early 1990s. We addressed this limitation through document analysis and interviews with key informants who closely collaborated with local government during this period.

While Cairns is currently experiencing a dengue‐free period, ongoing mosquito‐borne disease threats remain, including the potential introduction of *Ae. albopictus* from the nearby Torres Strait region.^51^ This could reignite dengue risk in Cairns and potentially impact the World Mosquito Programs success. With state and local government authorities now focusing less on checking for larval habitats in and around people's homes, there is potential for community complacency. Future research should explore the community's current perspectives on *Aedes* mosquito risk and preventative measures. This is pertinent in light of potential mixed messages from different initiatives advocating contradictory actions regarding mosquito control (e.g., encouraging mosquitoes to be released into the community, through the World Mosquito Program versus traditional government‐led messages aimed at preventing mosquito breeding). There may also be opportunities for TPHS to explore ways to involve the Cairns community to assist in *Wolbachia* mosquito monitoring or surveillance. With a growing number of citizen science mosquito surveillance strategies in Australia,^52^ a similar approach could be adopted to assist authorities and to keep mosquito‐borne disease risk on the community's radar.

## 5 CONCLUSION

With *Aedes* mosquito‐borne risk increasing in both high‐income and low‐income countries, it is of interest to understand what community engagement approaches have been used in areas that have historically experienced *Aedes*‐borne disease risk, and to look at reasons why these approaches are used. This study uniquely describes the various interconnecting factors influencing decisions around how the community is engaged and for what purpose. These findings offer insights that can guide future decision‐making in similar tropical settings, specifically by shedding light on key barriers such as resource constraints, regulations and institutional norms that may hinder the adoption of more empowering engagement approaches. Understanding these barriers can provide a foundation for identifying strategies to improve community engagement practices.

## FUNDING INFORMATION

Stephanie M. Topp holds a National Health and Medical Research Council Investigator Award, GNT1173004. No direct funding was provided for the design, implementation, or writing of the manuscript.

## CONFLICT OF INTEREST STATEMENT

The authors declare that they have no competing interests.

## ETHICS STATEMENT

The Townsville Hospital and Health Service HREC Human Research Ethics Committee, Australia, approved this study (HREC/2019/QTHS/53053). Interviews and subsequent analysis followed the relevant guidelines and regulations stipulated in the approval. The authors obtained informed, written consent from all the participants. Participation in the study was voluntary and confidential. Standards for Reporting Qualitative Research guided the reporting of this research.

## Supporting information


**Data S1.** Documents selected for analysis.

## Data Availability

James Cook University has a managed access system for data sharing that respects legal and ethical obligations to study participants to collect, manage and protect their data. Summarized non‐identified data supporting the conclusions of this article can be made available from the corresponding author (TA) upon reasonable request.
