# Supplementary material for: Factors influencing community engagement approaches used in Aedes aegypti management in Cairns, Australia
Source: Health Promot J Austr. 2024 Sep 25;36(2):e924. doi: 10.1002/hpja.924 (PMC11806404; doi:10.1002/hpja.924)
Supplement: Supplementary file 1 — Data S1. Documents selected for analysis. [file HPJA-36-0-s001.docx]

| **Supplementary 1 - Documents Selected for Analysis** |
| --- |
| **Grey Literature** |
| 1. Cairns Regional Council. Community Engagement Policy. (2018). Retrieved from: <https://www.cairns.qld.gov.au/__data/assets/pdf_file/0004/9184/Community_Engagement_General_Policy.pdf> |
| 1. Cairns Regional Council. Vector Control Unit. (2020). Retrieved from: https://www.cairns.qld.gov.au/community-environment/animals/mosquito-vector-control. |
| 1. Communicable Diseases Network Australia. (2015). National Guidelines for Public Health Units. Retrieved from: https://www.health.gov.au/sites/default/files/documents/2020/02/dengue-cdna-national-guidelines-for-public-health-units.pdf |
| 1. Mosquito Control Association of Australia. (Date Unknown). Public Relations Associated with Mosquito-borne Disease. In: Australian Mosquito Control Manual. (Unpublished Version) |
| 1. Public Relations Institute of Australia. (2004). Golden Target Awards: Dengue Blitz 04 - Dengue Fever Public Education Campaign Application. (Unpublished). |
| 1. Queensland Government. (2010). Queensland Joint Strategic Framework for Mosquito Management 2010-2015. Retrieved from: https://www.health.qld.gov.au/__data/assets/pdf_file/0019/444421/joint-strat-fwk.pdf |
| 1. Queensland Government. (2014). Queensland Chikungunya Management Plan 2014-2019. Brisbane. Retrieved from: <https://www.health.qld.gov.au/__data/assets/pdf_file/0027/444672/chikungunya-management-plan.pdf> |
| 1. Queensland Government. Queensland Dengue Management Plan. Brisbane.   Versions reviewed - 2000-2005; 2005-2010; 2015-2020 |
| 1. Queensland Government. Dengue - Queensland Health Guidelines for Public Health Units. Brisbane. Retrieved from: https://www.health.qld.gov.au/cdcg/index/dengue#pm |
| 1. Queensland Government. Queensland Public Health Act 2005. Retrieved from: https://www.legislation.qld.gov.au/view/pdf/inforce/current/act-2005-048. |
| 1. Queensland Government. Queensland Public Health Regulation 2018. (2022). Retrieved from: https://www.legislation.qld.gov.au/view/pdf/inforce/current/sl-2018-0117. |
| 1. Queensland Government. (Queensland Health). (1999). Dengue Fever Communications Plan. (Unpublished) |
| 1. Queensland Government. (Queensland Health). (2016). Public Health Practice Manual. Brisbane. |
| 1. Queensland Government. (Queensland Health). Dengue Fever Outbreaks. Retrieved from: <https://www.health.qld.gov.au/clinical-practice/guidelines-procedures/diseases-infection/diseases/mosquito-borne/dengue/dengue-outbreaks> |
| 1. The Local Government Association of Queensland. (2014). Mosquito Management Code of Practice. Retrieved from: <https://www.des.qld.gov.au/policies?a=272936:policy_registry/pr-cp-mosquito-management.pdf> |
| 1. World Mosquito Program. (2021) Cairns and surrounds. Retrieved from: <https://www.worldmosquitoprogram.org/en/global-progress/australia/cairns-and-surrounds> |
| 1. National Arbovirus and Malaria Advisory Committee. (2015) Framework for the surveillance, prevention and control of dengue virus infection in Australia. Retrieved from: <https://www.health.gov.au/sites/default/files/documents/2020/02/dengue-cdna-national-guidelines-for-public-health-units-framework-for-surveillance-prevention-and-control-of-dengue-virus-in-australia.pdf> |
| **Peer Review Publications** |
| 1. Spark, R., Sinclair, D., Donovan, R., Hanna, J., & Whitehead, K. Campaigns that bite: the dengue fever prevention campaign in far north Queensland. *Health Promotion Journal of Australia* 1994*;* 2, 28-32. |
| 1. Ritchie, S. A., Hanna, J. N., Hills, S. L., et al. Dengue control in North Queensland, Australia: case recognition and selective indoor residual spraying. *Dengue Bulletin 2002.*26. |
| 1. Hoffmann, A., Montgomery, B., Popovici, J., et al. Establishment of *Wolbachia* in *Aedes* populations to suppress dengue transmission. *Nature* 2011;476,454–457. <https://doi.org/10.1038/nature10356>. |
